# O brother, where art thou? Investment in siblings for inclusive fitness benefits, not father absence, predicts earlier age at menarche

**DOI:** 10.1098/rsbl.2017.0464

**Published:** 2017-10-18

**Authors:** Daniel Smith

**Affiliations:** Bristol Medical School: Population Health Sciences, University of Bristol, Bristol BS8 2BN, UK

**Keywords:** inclusive fitness, siblings, father absence, menarche, life history, ALSPAC

## Abstract

Numerous studies have indicated that father absence is associated with earlier age at menarche, with many evolutionary theories assuming that father absence is a causal factor that accelerates reproductive development. However, an alternative interpretation suggests that offspring may reproduce earlier in the presence of half- or step-siblings as the indirect fitness benefits to investing in them are lower, relative to delaying reproduction and investing in full siblings. From this perspective, father absence may perform no causal role in facilitating the onset of menarche. Using data from the Avon Longitudinal Study of Parents and Children, I find that individuals with only half- or step-siblings reach reproductive age earlier than those with only full siblings, with no independent effect of father absence. These results suggest that inclusive fitness benefits to investing in siblings, rather than father absence, may predict variation in age at menarche. These results provide a greater understanding of the adaptive mechanisms involved in reproductive decision-making, as well as potential implications for human life-history evolution and cooperative breeding more broadly.

## 1. Introduction

The onset of female reproductive potential—menarche—is an important stage in women's development, resulting in profound biological and socio-cultural change [1], yet the underlying evolutionary reasons for variation in reproductive development remain unclear. A large body of research has indicated that father absence is associated with younger age at menarche [2–5]. These findings present an evolutionary puzzle, as decreased parental investment, such as resulting from an absent father, ought to negatively impact offspring fitness [6], meaning that delayed reproduction may instead be expected.

One set of adaptive explanations, grouped under a ‘predictive adaptive response’ paradigm [7], suggests that individuals adapt their reproductive strategy when their father is absent to maximize future reproductive success. These theories posit that father absence may cue for a future environment in which it may be fitness-enhancing to reproduce earlier. Several variations of this general theory exist, including father absence as a cue for increased adult mortality [8] and father absence as an indicator of increased early-life adversity, which may predict a harsher adult environment [9]. However, these predictive adaptive response theories require that early-life environments are highly correlated with future environments, which may not be a plausible assumption in many cases [10]. A further theory suggests that father absence may result in reduced parental investment, causing children to invest in earlier reproduction rather than continued growth [2,3].

However, an alternative adaptive theory based on inclusive fitness considerations [11] suggests that these effects may not be driven by father absence, but rather differential indirect fitness benefits to investing in siblings as a function of relatedness [6,12]. Individuals may be more likely to forego immediate reproduction and invest in siblings if these are full siblings (*r* = 0.5) as the indirect fitness benefits are greater, relative to investing in half-siblings (*r* = 0.25; or *r* = 0 in the case of step-siblings). Indeed, previous research has indicated that the presence of half- or step-brothers is associated with earlier age at menarche [13]. From this perspective, father absence performs no causal role in facilitating the onset of menarche, but rather alters the relatedness between an individual and their subsequent siblings [6].

I, therefore, aim to explore the relative merits of each of these adaptive theories for the observed accelerated reproductive timing associated with father absence. According to predictive adaptive response or parental investment theories in which father absence performs a causal role in accelerating menarche, effects of half- or step-siblings ought to be independent of father absence. By contrast, theories based on inclusive fitness predict that the presence of half- or step-siblings may mediate the impact of father absence on age at menarche as children invest more in their own reproduction rather than their siblings' fitness. I find support for inclusive fitness considerations driving these results, as children with only full siblings reach menarche later than those with only half- or step-siblings, with no independent effect of father absence.

## 2. Material and methods

Data were obtained from the Avon Longitudinal Study of Parents and Children (ALSPAC) [14]. ALSPAC recruited 14 541 pregnant women resident in the former Avon Health Authority in southwest England with an estimated date of delivery between 1st April 1991 and 31st December 1992. When the oldest children were approximately 7 years of age an attempt was made to increase the initial sample with eligible cases who failed to join the original study. The total cohort is therefore 15 247 pregnancies, resulting in 15 468 fetuses, of which 14 701 were alive at 1 year of age (for more details see the cohort profile paper [14]).

Age at menarche was assessed from a series of nine questionnaires, completed approximately annually between the ages of eight and 17, and two research clinics attended at ages 13 and 16. First-reported age at onset of menarche was the dependent variable (for additional details, see [4]). Detailed family composition data obtained from a questionnaire completed by the child's mother (when the child was age seven) were used to assess relatedness between the study child and their siblings. Reproductive histories for each of the mother's relationships and those of their partner were ascertained, from which relatedness between the study child and siblings was determined. Four categories were constructed: only full siblings; only half- or step-siblings; both full and half/step-siblings; and no siblings. Half- and step-siblings were grouped together for practical reasons owing to small sample sizes (few individuals had only half- or step-siblings).

Father absence and the child's age when the biological father left were queried in three maternal questionnaires when the study child was aged seven, eight and ten. Any children whose father left prior to their sixth birthday were coded as ‘father absent’, while all other cases were coded as ‘father present’ (even if the father left at an older age). This cut-off was chosen as previous research with this sample has indicated that age at menarche in children with an absent father after age five is no different from those with a father present [4]. Potentially confounding variables were also assessed, including: birthweight, mother's highest education level, presence of severe financial problems, home ownership status (all of which may cue early-life adversity and socio-economic disadvantage), total number of siblings [15] and mother's self-reported age at menarche. Other than total number of siblings, control variables were collected during the mother's pregnancy. Descriptive statistics for all independent variables are displayed in electronic supplementary material, tables S1 and S2.

The total number of cases for which age at menarche, sibling relatedness and father absence data were available was 2921, which reduced to 2297 once other confounders were included. Please note that the study website contains details of all the data that are available through a fully searchable data dictionary (http://www.bristol.ac.uk/alspac/researchers/access/). Statistical analyses were conducted using multivariate linear regression models using the function *regress* in Stata v.14 (StataCorp., USA).

## 3. Results

**Figure 1.**
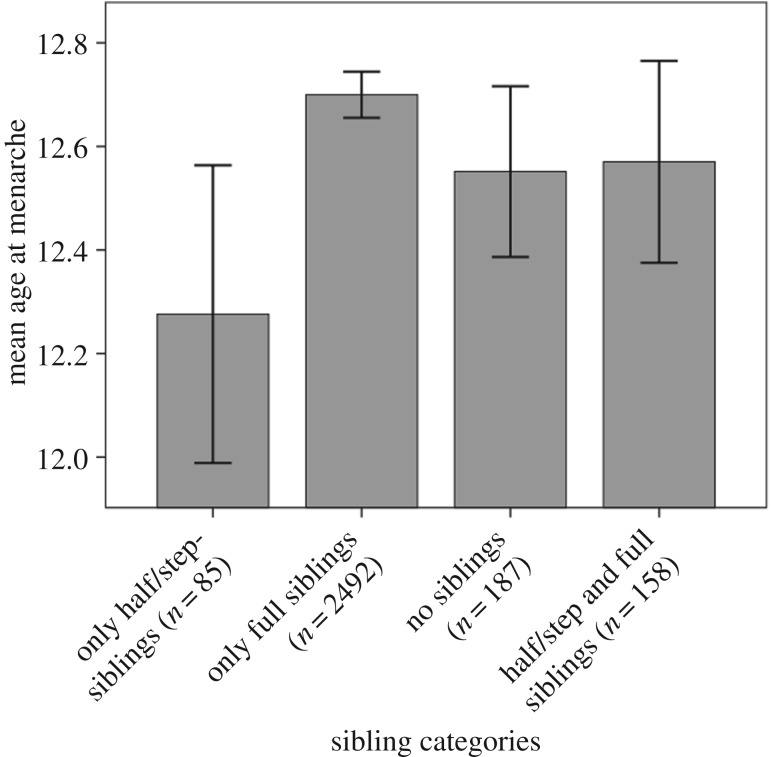
Mean age at menarche for each of the categories of sibling relatedness. Error bars denote 95% CI.

The average age at menarche in this sample was 12.62 (s.d. = 1.17), with notable differences between sibling categories (figure 1; electronic supplementary material, table S3). The average age at menarche for individuals with only half/step-siblings was 12.28 (s.d. = 1.33), while for those with only full siblings it was 12.7 (s.d. = 1.14). In a univariate model with ‘only full siblings’ as the reference group, there is strong evidence that age at menarche was lower in those with only half/step-siblings (*b* = −0.42, 95% CI:[−0.18; −0.67], *p* = 0.001, *d* = 0.37, *r*^2^ = 0.033; table 1, model 1). Consistent with previous research [4], in a univariate model containing just father absence, individuals with an absent father reached menarche at an earlier age (*b* = −0.23, 95% CI:[−0.12; −0.33], *p* < 0.001, *d* = 0.2, *r*^2^ = 0.01; table 1, model 2). In a model containing both ‘sibling relatedness’ and ‘father absence’, age at menarche was younger in those with only half/step-siblings (*b* = −0.37, 95% CI:[−0.11; −0.63], *p* = 0.005, *d* = 0.31, *r*^2^ = 0.023; table 1, model 3), with no independent effect of father absence (*b* = −0.11, 95% CI:[0.06; −0.27], *p* = 0.206).

**Table 1.** Models exploring the association between age at menarche and sibling relatedness. Model 1 presents the univariate model between age at menarche and sibling relatedness. Model 2 presents a univariate analysis of father absence and age at menarche. Model 3 combines sibling relatedness and father absence in the same model. Model 4 includes all potentially confounding variables. Model 5 presents an optimized version of the full model, retaining only variables associated with age at menarche (*p* < 0.1). For parameter estimates for the additional confounding variables in Models 4 and 5, see electronic supplementary material, table S4. 95% CI are displayed in brackets. *P-*value codes: ^†^<0.1; **<0.05; ***<0.01; ^#^<0.001.

variable	Model 1 (*n* = 2922)	Model 2 (*n* = 3743)	Model 3 (*n* = 2921)	Model 4 (*n* = 2297)	Model 5 (*n* = 2545)
sibling relationship (ref: only full siblings)					
only half/step-siblings	−0.42 [−0.18; −0.67]***	—	−0.37 [−0.11; −0.63]**	−0.38 [−0.1; −0.66]**	−0.4 [−0.15; −0.65]**
no siblings	−0.15 [0.02; −0.32]^†^	—	−0.13 [0.04; −0.3]	−0.2 [0.01; −0.41]^†^	−0.23 [−0.05; −0.4]*
half/step- and full siblings	−0.13 [0.05; −0.31]	—	−0.09 [0.1; −0.29]	0.06 [0.29; −0.18]	−0.04 [0.15; −0.23]
father absence (ref: no father absence)	—	−0.23 [−0.12; −0.33]***	−0.11 [0.06; −0.27]	−0.14 [0.05; −0.33]	—
additional confounding variables	no	no	no	yes^a^	yes^b^

^a^Additional confounding variables in model: birthweight, mother's highest education level, presence of severe financial problems, home ownership status, mother's age at menarche and total number of siblings.

^b^Additional confounding variables in model: mother's highest education level and mother's age at menarche.

These patterns remain if potentially confounding variables are included (table 1, model 4), as well as in an optimized model with non-predictive variables (*p* > 0.1) removed (table 1, model 5). In this optimized model, there was also evidence that individuals with no siblings likewise had an earlier age of menarche relative to those with only full siblings (*b* = −0.23, 95% CI:[−0.05; −0.4], *p* = 0.012, *d* = 0.21, *r*^2^ = 0.011), although the effect size is weaker compared to those with only half/step-siblings (*b* = −0.4, 95% CI:[−0.15; −0.65], *p* = 0.002; *d* = 0.36, *r*^2^ = 0.032). Collinearity between sibling relatedness and each of the independent variables is explored in electronic supplementary material, tables S5–S9; although some collinearity is present, variance inflation factors [16] from the regression models indicate that this is unlikely to bias parameter estimates (electronic supplementary material, table S10).

## 4. Discussion

These findings indicate that the inclusive fitness benefits to investing in siblings, rather than father absence, predict variation in age at menarche. Individuals with only half- or step-siblings were found to reach reproductive age on average five months earlier than those with only full siblings (figure 1). This is consistent with individuals investing more in their siblings if they are full siblings for indirect fitness benefits, while individuals with half- or step-siblings are more likely to invest in their own reproduction as the inclusive fitness gains are lower [6,12]. One plausible mechanism driving these findings is ‘intergenerational reproductive conflict’ [17]. If there is reproductive competition between mothers and children (such that competing for reproductive resources damages the other's inclusive fitness), children are more likely to invest in their mother's reproduction (i.e. delay reproduction and invest in siblings) in the presence of full, rather than half- or step-, siblings [6].

Controlling for the relationship between siblings, no independent effect of father absence was observed. Father absence may therefore play no causal role in accelerating the onset of reproductive potential. Although these results provide evidence against ‘father absence’ hypotheses, other forms of early-life adversity or high levels of extrinsic mortality may still influence reproductive decision-making, consistent with life-history theory [2,18]. Here, I only demonstrate that father absence may not accurately represent these adaptive challenges.

These results also have wider implications for understanding the evolution of human life history. They suggest that the evolution of extended childhood and cooperative breeding in humans [19,20] may in part be owing to long-term pair-bonding resulting in inclusive fitness gains to investing in full siblings. Ethnographic studies have indicated that a significant proportion of allomaternal care is from siblings [21] and that the presence of older siblings often increases offspring survival [22], suggesting that cooperation occurs between siblings and has important fitness consequences. These patterns are also found in industrial societies, where young siblings engage in repeated cooperative interactions [23], while among adults full siblings invest more in one another than half-siblings [24]. These findings are consistent with comparative phylogenetic analyses demonstrating that monogamous mating systems, resulting in increased relatedness between siblings, preceded the evolution of cooperative breeding in birds [25], mammals [26] and eusocial insects [27]. Although requiring additional research, these findings suggest that the evolution of extended childhood in humans may, in part, be owing to kin selected benefits of cooperating with full siblings.

## Supplementary Material

Supplementary Material

## Supplementary Material

Analysis Code

## Data accessibility

Data used for this submission will be made available on request to the Executive (alspac-exec@bristol.ac.uk). The ALSPAC data management plan (available here: http://www.bristol.ac.uk/alspac/researchers/data-access/documents/alspac-data-management-plan.pdf) describes in detail the policy regarding data sharing, which is through a system of managed open access. Stata code supporting this article has been uploaded as part of the electronic supplementary material.
