# Supplementary material for: O brother, where art thou? Investment in siblings for inclusive fitness benefits, not father absence, predicts earlier age at menarche
Source: Biol Lett. 2017 Oct 18;13(10):20170464. doi: 10.1098/rsbl.2017.0464 (PMC5665773; doi:10.1098/rsbl.2017.0464)
Supplement: Supplementary Material [file rsbl20170464supp1.docx]

Supplementary Material for ‘O Brother, where art thou? Investment in siblings for inclusive fitness benefits, not father absence, predicts earlier age at menarche’

Author: Daniel Smith

*Table S1:* Descriptive statistics for binary or categorical independent variables, for cases where there is both age at menarche and sibling relatedness data.

| **Variable (*n*)** |
| --- |
| *Sibling Relatedness* (*n*=2,922) |
| Only half/step siblings (*n*=85) |
| Only full siblings (*n*=2,492) |
| No siblings (*n*=187) |
| Half/step siblings and full siblings (*n*=158) |
|  |
| *Father Absence when Study Child was Age 5 or Younger* (*n*=2,921) |
| Father absence (*n*=245) |
| No father absence (*n*=2,676) |
|  |
| *Use of Car by Mother or Partner* (*n*=2.854) |
| Yes (*n*=2,738) |
| No (*n*=116) |
|  |
| *Mother’s Highest Educational Qualification* (*n*=2,859) |
| Certificate of Secondary Education (CSE; *n*=339) |
| Vocational qualification (*n*=215) |
| O level (*n*=1,014) |
| A level (*n*=764) |
| Degree (*n*=527) |
|  |
| *Home Ownership Status* (*n*=2,858) |
| Owned (*n*=2,433) |
| Rented (*n*=425) |
|  |
| *Major Financial Problems* (*n*=2,661) |
| No (*n*=2,407) |
| Yes (*n*=254) |

*Table S2:* Descriptive statistics for continuous independent variables, for cases where there is both age at menarche and sibling relatedness data.

| **Variable (*n*)** | **Mean (SD)** |
| --- | --- |
| Birthweight (grams; *n*=2,885) | 3379.22 (506.05) |
| Mother’s Age at Menarche (*n*=2,584) | 12.8 (1.49) |
| Total Number of Siblings (*n*=2,922) | 1.45 (0.86) |

*Table S3:* Descriptive statistics for age at menarche, split by sibling relatedness categories (see also figure 1).

| **Sibling Relatedness (*n*)** | **Mean Age at Menarche (SD)** |
| --- | --- |
| Only half/step-siblings (*n*=85) | 12.28 (1.33) |
| Only full siblings (*n*=2,492) | 12.7 (1.14) |
| No siblings (*n*=187) | 12.55 (1.14) |
| Half/step siblings and full siblings (*n*=158) | 12.57 (1.24) |
| Full sample (*n*=4,146) | 12.62 (1.17) |

*Table S4:* Expanded table displaying the ‘additional confounding variables’ in models 4 and 5 from table 1. 95% confidence intervals are displayed in brackets. *P-*value codes: **˙**<0.1; *<0.05; **<0.01; ***<0.001.

| **Variable** | **Model 4 (*n*=2,297)** | **Model 5 (*n*=2,545)** |
| --- | --- | --- |
| **Sibling relationship (ref: Only full siblings)** |  |  |
| **Only half/step-  siblings** | -0.38 [-0.1; -0.66]** | -0.4 [-0.15; -0.65]** |
| **No siblings** | -0.2 [0.01; -0.41] **˙** | -0.23 [-0.05; -0.4]* |
| **Half/step and full   siblings** | 0.06 [0.29; -0.18] | -0.04 [0.15; -0.23] |
| **Father absence (ref: No father absence)** | -0.14 [0.05; -0.33] | - |
| **Birthweight (grams)** | 0.00003 [0.00012; -0.00006] | - |
| **Mother’s highest education level (ref: CSE)** |  |  |
| **Vocational** | -0.03 [0.18; -0.25] | -0.01 [0.19; -0.21] |
| **O level** | 0.13 [0.29; -0.02] **˙** | 0.15 [0.01; 0.3]* |
| **A level** | 0.19 [0.03; 0.35]* | 0.22 [0.07; 0.37]** |
| **Degree** | 0.14 [0.32; -0.03] | 0.17 [0.01; 0.33]* |
| **Home ownership status (ref: Owned)** | -0.05 [0.08; -0.19] | - |
| **Major financial problems (ref: No)** | -0.06 [-0.09; 0.22] | - |
| **Mother’s age at menarche** | 0.23 [0.2; 0.26]*** | 0.22 [0.19; 0.25] |
| **Total number of siblings** | -0.00 [0.07; -0.07] | - |

*Table S5:* Association between sibling relatedness and father absence. Although a *chi*-squared test indicates that children with only half- or step-siblings are more likely to have an absent Father (χ²(3, *n*=2,291)=439.3, *p*<0.001), variance inflation factors derived from the regression models indicate that this collinearity is unlikely to bias parameter estimates (table S11). Row percentages are presented in brackets.

|  | **No Father Absence** | **Father Absence** |
| --- | --- | --- |
| **Only Half/Step-Siblings** | 45 (53.6%) | 39 (46.4%) |
| **Only Full Siblings** | 2,387 (95.8%) | 105 (4.2%) |
| **No Siblings** | 146 (78.1%) | 41 (21.9%) |
| **Half/Step and Full Siblings** | 90 (62%) | 60 (38%) |
| **Total** | 2,676 (91.6%) | 245 (8.4%) |

*Table S6:* Association between sibling relatedness and family use of a car. A *chi*-squared test indicates that children with only half- or step-siblings are less likely to have access to a car (χ²(3, *n*=2,854)=90.69, *p*<0.001). Row percentages are presented in brackets.

|  | **Access to Car** | **No Access to Car** |
| --- | --- | --- |
| **Only Half/Step-Siblings** | 67 (80.7%) | 16 (19.3%) |
| **Only Full Siblings** | 2,370 (97.1%) | 71 (2.9%) |
| **No Siblings** | 170 (95%) | 9 (5%) |
| **Half/Step and Full Siblings** | 131 (86.8%) | 20 (13.3%) |
| **Total** | 2,738 (95.9%) | 116 (4.1%) |

*Table S7:* Association between sibling relatedness and Mother’s highest education qualification. A *chi*-squared test indicates that children with only half- or step-siblings are less likely to have Mother’s with a high education level (χ²(12, *n*=2,859)=98.86, *p*<0.001). Row percentages are presented in brackets.

|  | **CSE** | **Vocational** | **O Level** | **A Level** | **Degree** |
| --- | --- | --- | --- | --- | --- |
| **Only Half/Step-Siblings** | 17 (20.5%) | 11 (13.3%) | 34 (41%) | 12 (14.5%) | 9 (10.8%) |
| **Only Full Siblings** | 263 (10.7%) | 173 (7.1%) | 867 (35.4%) | 660 (27%) | 485 (19.8%) |
| **No Siblings** | 16 (8.9%) | 12 (6.7%) | 57 (31.8%) | 69 (38.6%) | 25 (14%) |
| **Half/Step and Full Siblings** | 43 (28.9%) | 19 (12.8%) | 56 (37.6%) | 23 (15.4%) | 8 (5.4%) |
| **Total** | 339 (11.9%) | 215 (7.5%) | 1,014 (35.5%) | 764 (26.7%) | 527 (18.4%) |

*Table S8:* Association between sibling relatedness and home ownership status. A *chi*-squared test indicates that children with only half- or step-siblings are more likely to live in rented accommodation (χ²(3, *n*=2,858)=167.37, *p*<0.001). Row percentages are presented in brackets.

|  | **Own Accommodation** | **Rented Accommodation** |
| --- | --- | --- |
| **Only Half/Step-Siblings** | 46 (54.8%) | 38 (45.2%) |
| **Only Full Siblings** | 2,140 (87.6%) | 302 (12.4%) |
| **No Siblings** | 159 (88.8%) | 20 (11.2%) |
| **Half/Step and Full Siblings** | 88 (57.5%) | 65 (42.5%) |
| **Total** | 2,433 (85.1%) | 425 (14.9%) |

*Table S9:* Association between sibling relatedness and major financial problems. A *chi*-squared test indicates that children with only half- or step-siblings are more likely to have experienced major financial difficulties (χ²(3, *n*=2,858)=167.37, *p*<0.001). Row percentages are presented in brackets.

|  | **No Financial Problems** | **Financial Problems** |
| --- | --- | --- |
| **Only Half/Step-Siblings** | 63 (84%) | 12 (16%) |
| **Only Full Siblings** | 2,072 (91.3%) | 198 (8.7%) |
| **No Siblings** | 151 (87.3%) | 22 (12.7%) |
| **Half/Step and Full Siblings** | 121 (84.6%) | 22 (15.4%) |
| **Total** | 2,407 (90.5%) | 254 (9.6%) |

*Table S10:* One-way Analysis of Variance (ANOVA) between sibling relatedness and continuous independent variables (birthweight, mother’s age at menarche and total number of siblings). No associations between sibling relatedness and birthweight or mother’s age at menarche were reported, while total number of siblings was associated with sibling relatedness; unsurprisingly, individuals with no siblings had fewer siblings, while individuals with both half/step and full siblings possessed a greater total number of siblings.

| **Sibling Relatedness Category** | **Mean Birthweight (grams: SD)** | **Mean Mother’s Age at Menarche (SD)** | **Mean Total Number of Siblings (SD)** |
| --- | --- | --- | --- |
| **Only Half/Step-Siblings** | 3374.64 (522.1) | 12.84 (1.68) | 1.62 (0.84) |
| **Only Full Siblings** | 3379.12 (504.21) | 2,195 (12.81) | 1.46 (0.7) |
| **No Siblings** | 3349.94 (481.17) | 12.76 (1.46) | 0 (0) |
| **Half/Step and Full Siblings** | 3417.82 (555.25) | 12.76 (1.53) | 2.94 (1.16) |
| ***F*-Statistic and *p*-value** | *F*(3, 2,881)=0.51; *p*=0.677 | *F*(3, 2,580)=0.12; *p*=0.949 | *F*(3, 2,918)=524.44; *p*<0.001 |

*Table S11:* Variance inflation factors (VIFs) to test for collinearity in each of the multivariate models from table 1 (models 3, 4 and 5). A VIF greater than three is indicative of high collinearity, which may bias parameter estimates. None of the VIFs reported here are greater than 3, indicating that collinearity between predictor variables is unlikely to bias these results.

| **Variable** | **Model 3 VIFs** | **Model 4 VIFs** | **Model 5 VIFs** |
| --- | --- | --- | --- |
| **Sibling relationship (ref: Only full siblings)** |  |  |  |
| **Only half/step-  siblings** | 1.08 | 1.12 | 1.01 |
| **No siblings** | 1.04 | 1.31 | 1.01 |
| **Half/step and full   siblings** | 1.1 | 1.36 | 1.01 |
| **Father absence (ref: No father absence)** | 1.18 | 1.21 | - |
| **Birthweight (grams)** | - | 1 | - |
| **Mother’s highest education level (ref: CSE)** |  |  |  |
| **Vocational** | - | 1.55 | 1.52 |
| **O level** | - | 2.78 | 2.68 |
| **A level** | - | 2.62 | 2.5 |
| **Degree** | - | 2.27 | 2.19 |
| **Home ownership status (ref: Owned)** | - | 1.11 | - |
| **Major financial problems (ref: No)** | - | 1.02 | - |
| **Mother’s age at menarche** | - | 1.01 | 1 |
| **Total number of siblings** | - | 1.54 | - |
